# Enhancing prediction and inference of daily in-stream nutrient and sediment concentrations using an extreme gradient boosting based water quality estimation tool - XGBest

**DOI:** 10.1016/j.scitotenv.2025.178517

**Published:** 2025-01-20

**Authors:** Shubham Jain, Arun Bawa, Katie Mendoza, Raghavan Srinivasan, Rajbir Parmar, Deron Smith, Kurt Wolfe, John M. Johnston

**Affiliations:** a Water Management and Hydrological Science, Texas A&M University, College Station, TX, USA; b Texas A&M AgriLife Research, Blackland Research & Extension Center, Temple, TX, USA; c Office of Research and Development, United States Environmental Protection Agency, Athens, GA, USA

**Keywords:** Total nitrogen, Sediments, LOADEST, WRTDS, Machine learning, HAWQS

## Abstract

Estimating constituent loads in streams and rivers is a crucial but challenging task due to low-frequency sampling in most watersheds. While predictive modeling can augment sparsely sampled water quality data, it can be challenging due to the complex and multifaceted interactions between several sub-watershed eco-hydrological processes. Traditional water quality prediction models, typically calibrated for individual sites, struggle to fully capture these interactions. This study introduces XGBest, a machine learning-based tool, that integrates hydrological data, land cover, and physical watershed attributes at a regional scale to predict daily concentrations of Total Nitrogen (TN), Total Phosphorus (TP), and Total Suspended Solids (TSS). XGBest leverages 29 environmental variables, including daily and antecedent discharge, temporal features, and landscape characteristics, to comprehensively evaluate water quality dynamics across a large hydrologic region. To explore the robustness of the developed tool, XGBest was validated using observed water quality data in three different hydrologic regions in the eastern United States, encompassing 499 water quality monitoring sites characterized by diverse hydro-climatic conditions and watershed attributes. This study also employed the legacy United States Geological Survey (USGS) tools - LOADEST and WRTDS as benchmarks to evaluate the performance of XGBest in these regions.

The results demonstrated that XGBest outperformed LOADEST and WRTDS in predictive accuracy and revealed critical insights into the spatial and temporal variability of nutrient and sediment loads. In addition, SHapley Additive exPlanations (SHAP) values highlighted the importance of integrating static and dynamic watershed attributes, such as land cover, antecedent discharge, and seasonality, in capturing the complex concentration-discharge (C-Q) relationships. This study positions XGBest as a robust and scalable water quality prediction tool that bridges the gap between hydrology and broader environmental management. By combining multiple environmental factors into a unified predictive framework, XGBest enhances our understanding of water quality and supports more effective environmental monitoring and management strategies.

## Graphical Abstract

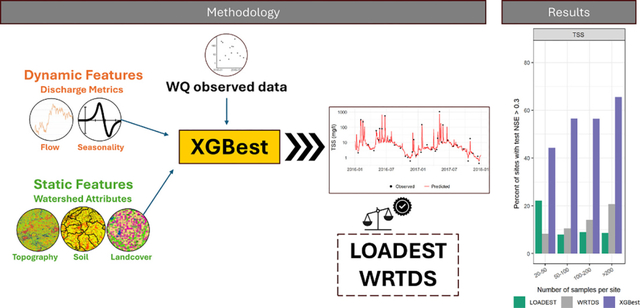

## 1. Introduction

Elevated concentrations of nutrients and sediments in surface waterbodies can present considerable threats to human health and ecological well-being (Dodds and Welch, 2000; Smith et al., 1999). Eutrophication is one of the primary causes of coastal and inland ecosystem degradation worldwide, leading to harmful algal blooms and dead zones in aquatic systems (Oelsner and Stets, 2019; Zhang et al., 2022). Considering the significant implications of water quality degradation, continuous and effective monitoring of instream constituents is critical for setting regulatory actions, assessing management and conservation efforts, and evaluating flux trends. However, as most water quality monitoring programs (WQMPs) typically involve lab analysis for measurement using in-situ ‘grab samples’ which is inherently an expensive task, water quality data are globally sparse, both spatially and temporally (Saha et al., 2023; Zhi et al., 2024). The lack of sufficient monitoring data can elevate uncertainty in decision-making, potentially leading to outcomes and trends that deviate from targeted goals (Zadeh et al., 2019).

Although there have been numerous attempts to estimate daily concentrations (C) of instream nutrients and sediments using discrete samples, most statistical approaches are limited to using site-specific discharge (Q), time (T), and season (S) as explanatory variables in a regression model, which includes the legacy United States Geological Survey (USGS) Load Estimator Tool (LOADEST) (Runkel et al., 2004), and the Weighted Regressions on Time, Discharge, and Season (WRTDS) tool (Hirsch et al., 2010). Understanding the concentration-discharge (C-Q) relationship and its changes over time is critical to identifying solute and particulate export regimes and patterns at the watershed outlet (Ebeling et al., 2021). The C-Q relationship can be broadly classified as either chemostatic (low variability of C compared to Q) or chemodynamic (high C-Q variability). The nature of the C-Q regime depends on the type of solute or particulate, watershed characteristics of discharge, and the sources and sinks of the solutes or particulates in the watershed. Moatar et al. (2017) showed nine possible C-Q modalities (e. g., flushing until median flow and then chemostatic, flushing until median flow, then dilution for high flows) when segmenting the hydrograph at the median discharge. However, only two or three modalities were dominant for most solutes analyzed in the study. Similarly, Saha et al. (2023) found four dominant patterns in the nitrate C-Q relationship when split at the median discharge (flushing, flushing and chemostatic, flushing and dilution, and chemodynamic).

The ability of statistical models to quantify seasonal or long-term trends in solute and particulate concentrations depends on the length of available sampling data and the complexity of the C-Q relationship (Saha et al., 2023). Regression-based approaches applied at a single sampling site require the sampling period and frequency to be much larger than often available. For example, the recommended sampling criteria for WRTDS is at least 20 years of data and the availability of >200 samples to conduct the analysis (Hirsch et al., 2010). However, the global average data count of total suspended solids (TSS), one of the most abundantly measured water quality parameters, is only 29 per station, with an average record length of 4.2 years (Zhi et al., 2024). Therefore, there is limited practicality in employing such methods for sites with insufficient monitoring data to capture the entire streamflow regime.

### Assumption I.

Aggregating observed water quality data across a hydrologic region (at 2-digit USGS Hydrologic Unit Code scale – HUC02 (USGS and USDA-NRCS, 2013)) can overcome the limitations of insufficient sampling data at individual sites, thereby improving prediction model accuracy.

Machine Learning (ML) algorithms have been increasingly applied in several hydrological applications, including water quality prediction (Zhi et al., 2024). As ML algorithms typically require a large training dataset, they can either be applied to sites with high-frequency concentration data (Saha et al., 2023) or at a regional scale by combining data from multiple monitoring stations (Adedeji et al., 2022; Alnahit et al., 2022; Basu et al., 2023; Isles, 2024). Saha et al. (2023) used the Long Short-Term Memory (LSTM) model to predict nitrate concentrations using high-frequency monitoring sites in Iowa and found that observations from neighboring sites were crucial in determining continuous daily nitrate concentration. However, LSTMs are not very useful for sites where monitoring data are only available at a monthly or quarterly temporal scale. Isles (2024) showed that Random Forests (RF) models outperformed WRTDS in predicting daily nutrient concentrations, possibly due to a more flexible representation of seasonality using data from 17 tributaries to Lake Champlain. However, their model did not include any representation of watershed attributes specific to each site.

### Assumption II.

ML techniques can reveal complex relationships between water quality constituent concentrations, discharge metrics, and watershed attributes, thereby improving prediction model accuracy.

Differences in watershed attributes (geometry, geology, topography, etc.) have been proven to translate well into identifying heterogeneous hydrologic behavior in cases of regional hydrological modeling, leading to improved predictions (Kratzert et al., 2019). In cases of water quality prediction, Basu et al. (2023), using data from >200 monitored streams in the Great Lakes Basin, showed that percentages of tile-drained area, developed land, wetland, and forested land were the top predictors of mean annual flow-weighted concentrations of Dissolved Inorganic Nitrogen (DIN) and Soluble Reactive Phosphorus (SRP). Similarly, Alnahit et al. (2022) found that urbanization and cropping were highly relevant to long-term median values of in-stream total nitrogen (TN) and total phosphorus (TP).

### Assumption III.

Incorporating watershed attributes (e.g., geometry, topography, geology) alongside discharge dynamics will improve the accuracy of water quality prediction models.

However, gathering these relevant watershed attributes for many water quality monitoring sites can be arduous. Although there are large sample hydrology datasets with watershed attributes available in the US such as CAMELS-US (Addor et al., 2017), HYSETS (Arsenault et al., 2020), and the USGS GAGES-II (Falcone, 2011), they are limited to watersheds that drain into a streamflow gaging station. On the other hand, the Hydrologic and Water Quality System (HAWQS) is a web-based decision support system that utilizes the Soil and Water Assessment Tool (SWAT) model as its core engine and covers the entire conterminous United States at USGS Hydrologic Unit Code (HUC) 8 (subbasin) to 14 (subwatershed) level resolution (HAWQS, 2023; Yen et al., 2016). Watershed attributes pre-defined over each monitoring site’s drainage area can be extracted from HAWQS for large sample hydrological studies FAIR-ly (Findable, Accessible, Interoperable, and Reproducible) (Wilkinson et al., 2016).

The primary objective of this study was to develop a robust water quality prediction tool while addressing aforementioned hypotheses and overcoming challenges associated with limited data availability. In this study, we introduce XGBest (eXtreme Gradient Boosting based water quality ESTimation), a machine learning-based tool using the Extreme Gradient Boosting regression approach, to estimate daily instream nutrients (Total Nitrogen, TN; Total Phosphorus, TP) and sediment (Total Suspended Solids, TSS) concentrations using discrete water quality samples and observed discharge along with static watershed attributes. This study also evaluates and validates XGBest’s predictive performance against observed water quality data, while benchmarking it against the two legacy United States Geological Survey (USGS) models: Load Estimator Tool (LOADEST) (Runkel et al., 2004) and the Weighted Regressions on Time, Discharge, and Season (WRTDS) tool (Hirsch et al., 2010). To test its broad applicability and robustness, XGBest was applied across three distinct hydrologic regions, covering a wide range of land cover and climatic conditions. Finally, we evaluate the feature-response relationships in the XGBest model using feature importance and partial dependence structures obtained from SHapley Additive exPlanations (SHAP).

## 2. Sites and data

### 2.1. Study area

**Fig. 1.**
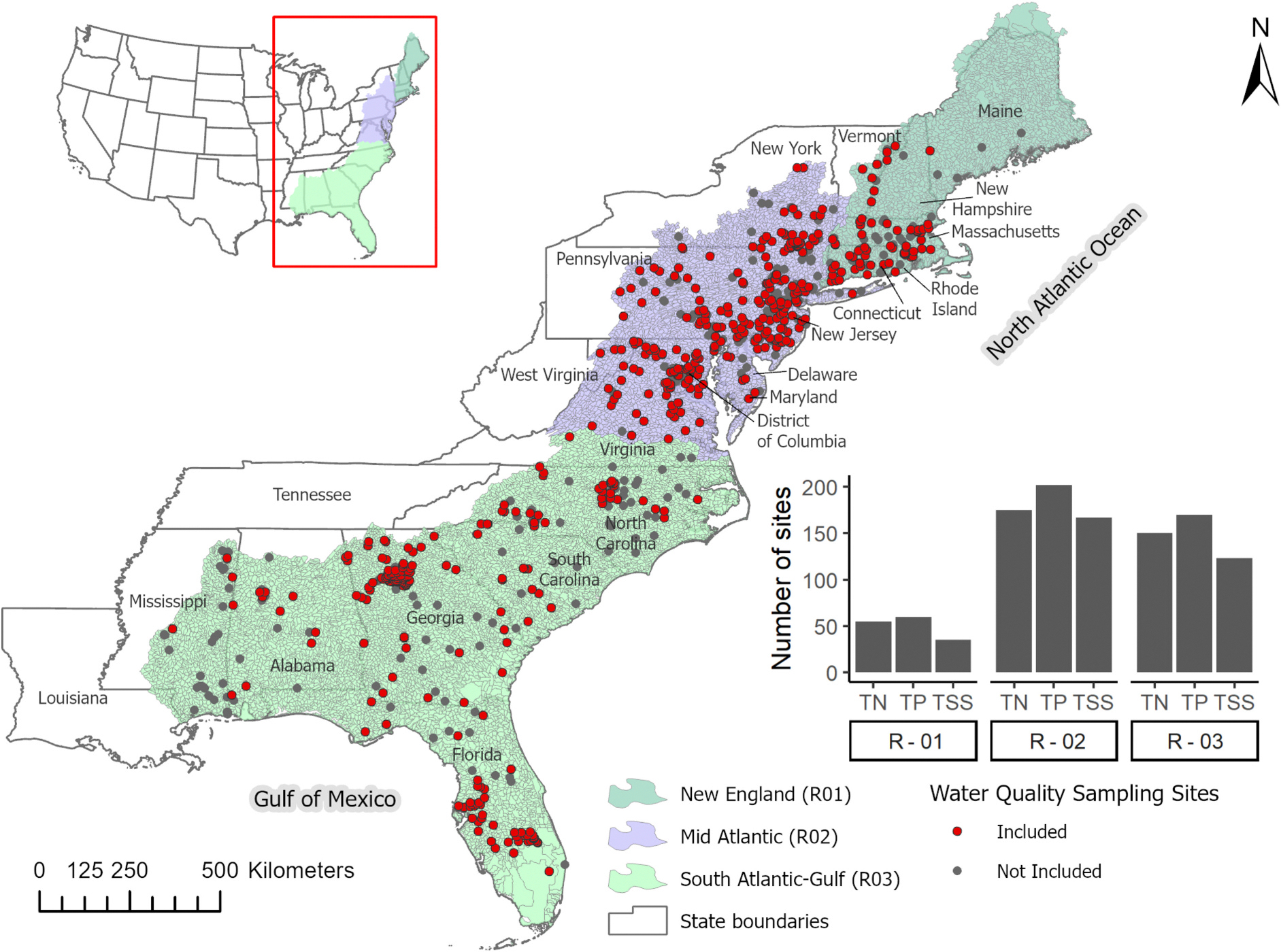
Location of the study area encompassing three hydrologic regions in the Eastern United States: New England, Mid-Atlantic, and South Atlantic-Gulf, including the locations of water quality sampling stations with available sampling data within the study period (1996–2020). The criteria for ‘*not included*’ water quality sites are detailed in Section 2.2.1. The bar plot (inset) illustrates the number of sites with samples for each water quality parameter within each hydrologic region.

This study focuses on aggregating water quality data within each HUC02 hydrologic region to train the XGBest model, thereby developing a single prediction model for each constituent within a region to predict constitute concentrations at individual site. We considered three USGS HUC02 regions in this study to evaluate and validate XGBest across varying land cover and climatic conditions: New England (Region – 01; R-01), Mid-Atlantic (Region – 02; R-02), and South-Atlantic Gulf (Region – 03; R-03) (Fig. 1). These regions include some of the major coastal and estuarine areas in the U.S. (e.g., the New York Bight, Chesapeake Bay, and Mobile Bay) with heightened water quality concerns and frequent hypoxic zones due to anthropogenic activities and the remobilization of legacy nutrients and sediments (Bricker et al., 2008; Chang et al., 2021). The Mid-Atlantic Region (R-02) is a major source of nutrient enrichment in Chesapeake Bay, the largest estuary in the United States (Zhang et al., 2022). Phosphorus (P) fluxes to the bay have been observed to increase from five of its nine major tributaries, including the Susquehanna River, the largest tributary to the bay (Zhang et al., 2023). However, nitrogen (N) fluxes to the Chesapeake Bay from seven of its nine major tributaries have shown decreasing trends since the early 1990s, largely driven by reduced point sources and atmospheric N deposition (Ator et al., 2020, 2019; Zhang et al., 2023). In contrast, N loads in the South Atlantic Gulf region (R-03) have not shown a decreasing nutrient trend in most sites that contribute to sensitive estuaries (Oelsner and Stets, 2019). Overall, these regions face similar water quality concerns that require improved methods for evaluating the spatial and temporal variability of nutrients and sediments, given the constraints of limited data availability.

### 2.2. Data sources and preparation

XGBest was trained using 29 features, including 6 dynamic features (discharge and seasonality metrics) and 23 static watershed attributes (based on land cover, topography, and geology). This subsection outlines the data sources, and preprocessing steps used to train and validate the XGBest tool.

#### 2.2.1. Water quality and discharge data: Response variables and dynamic features

Observed concentrations of nutrients (TN and TP) and sediments (TSS) for all study sites were obtained from the Water Quality Portal (Read et al., 2017), which contains US-based national-scale aggregated water quality records. The monitoring data were extracted for a 25-year study period between 01/01/1996 and 12/31/2020. The Daily discharge corresponding to each water quality monitoring site were obtained from the USGS National Water Information System (De Cicco et al., 2018). However, as not all sites had daily discharge records, the nearest gauge on the same stream segment as the monitoring site was used, and discharge values were adjusted using the drainage area ratio between the two locations (Asquith et al., 2006; Basu et al., 2023; Bawa et al., 2024). Further, sites were filtered based on the following criteria: (a) availability of at least 20 sampling days between 1996 and 2020, (b) availability of observed discharge records at the sampling location or a nearby location within the same stream segment, and (c) sites whose drainage area was completely within the conterminous U.S. boundary.

**Fig. 2.**
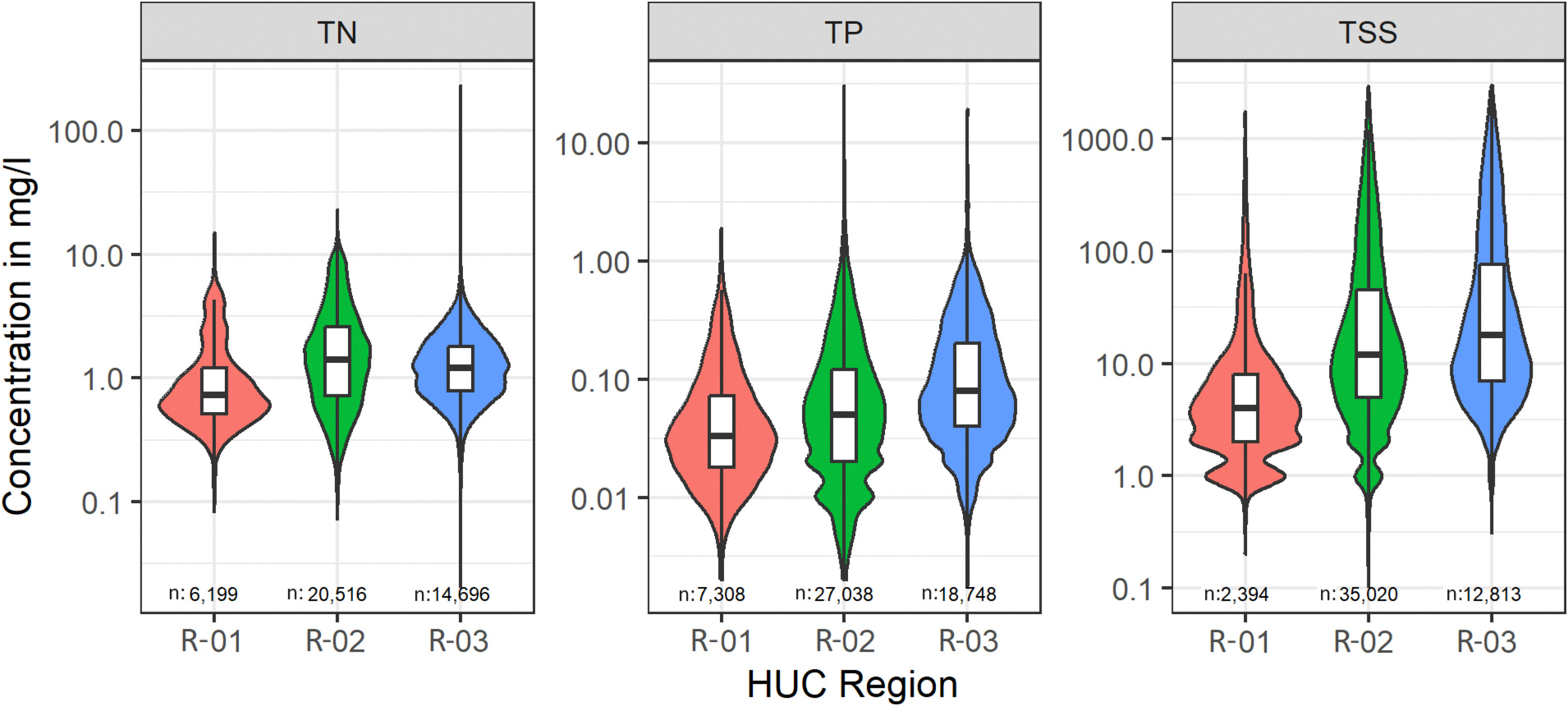
Boxplots of sampled values between the study period (1996–2020) for each water quality parameter (TN, TP, and TSS) categorized by the hydrologic region. The total number of samples for each water quality parameter within each region is indicated by *N*.

To remove outliers from the observed data to account for potential measurement errors, we calculated the Mahalanobis distances (Etherington, 2021; Uddin et al., 2024) between all samples using constituent concentration, discharge, and channel length and excluded the top 0.1 % of values with the largest deviation from the mean. A total of 499 sites containing nutrient and/or sediment data were selected for the study (TSS: 325 sites, TP: 432 sites, and TN: 380 sites). The median number of samples per site across all three regions were 89 for TN, 95 for TP, and 71 for TSS. The range of observed water quality constituent concentrations of selected samples across each hydrologic region is shown in Fig. 2, and the distribution of sample sizes within selected sites is available in the Supplementary Information (SI; Fig. S1).

In addition to the observed daily discharge (Q), the antecedent discharge variables were calculated to capture any hysteresis effects in the C-Q relationship, represented by ΔQ: 1-day lagged discharge, 7dQ: 7-day rolling average discharge, and 30dQ: 30-day rolling average discharge (Adedeji et al., 2022; Isles, 2024). Natural logarithms of these discharge variables were used in the XGBest model (Table 1). The decimal year (DecYear) and day of the year (DOY) corresponding to the observed discharge were also obtained to account for seasonal patterns and long-term trends in water quality.

#### 2.2.2. Watershed attributes: static features

To capture the spatial variability in water quality constituent concentrations, we selected 23 static watershed attributes, grouped into five major categories: land cover, hydrology, soil, sediment potential, and channel characteristics (Table 1). All static attributes, excluding the baseflow index (BFI), were derived using HAWQS input/output files for each HUC-12 scale subbasin within the three major hydrological regions (Bawa et al., 2024; HAWQS, 2023). BFI values at the HUC-12 scale were derived from Wolock (2003). Subsequently, the watershed attributes corresponding to the drainage area of each sampling site were estimated using an area-weighted average of the attributes from all contributing subbasins.

The nomenclature adopted for the watershed attributes in this study, along with their description, is provided in Table 1. The major land cover classes derived from HAWQS were grouped into five categories to reduce the number of features in the XGBest model: developed, forest, wetland, grassland/shrub, and managed vegetation. The soil type, land cover, and slope, in combination, directly governs the sediment loading potential of a watershed. Therefore, we categorized the watershed area into five sediment loading potential classes using watershed slope and curve number (CN) obtained from HAWQS: very high, high, moderate, low, and very low sediment loading potential (these categories are detailed in Table S1 in SI). The percentage of watershed area in each class was used as watershed attributes.

## 3. Methods

### 3.1. Extreme gradient boosting

#### 3.1.1. Model development

Extreme gradient boosting (XGB) is a tree-based boosting and supervised learning method proposed by Chen et al. (2016). The method employs an ensemble of sequentially constructed decision trees, where each subsequent tree is trained on the residuals of the previous ensemble, thereby iteratively minimizing the overall error of the model, which is also the primary distinction from the widely adopted Random Forest algorithm (Alnahit et al., 2022; Behrouz et al., 2022; Isles, 2024), in which each tree is constructed independently. XGB is particularly beneficial for water quality modeling due to its optimized handling of sparse and missing data and its ability to incorporate regularization to prevent overfitting.

The XGBest tool consists of a total of nine individual XGB models (three constitutes × three hydrologic regions) developed in this study for predicting the natural logarithm of daily constituent concentrations (ln (C)) by aggregating data from all sites within a HUC02 region. To improve the robustness of the trained model, the hyperparameters for XGBest were selected using an automated grid search and 5-fold cross-validation, performed during model training. The hyperparameters for XGB regression include max*imum tree depth*, learning rate (*eta*), regularization term (*gamma*), the number of samples and attributes supplied to the tree (*subsample* and *colsample_bytree*), *minimum child node weight*, and *max delta step*. The *maximum tree depth* limits the depth of each decision tree, controlling the model complexity. The regularization term (*gamma*) prevents overfitting by penalizing the complexity of trees. The *subsample* and *colsample_bytree* hyperparameters control the fraction of data randomly sampled for each tree, reducing overfitting by ensuring diverse and less correlated trees. The range of possible values used in the grid search and the final hyperparameter values for the nine models are provided in the SI (Table S2).

*Re*-transformation of the predicted ln(C) values to the original units can introduce bias in the final estimates of the constituent concentrations (Cohn et al., 1989; Duan, 1983). To correct for this re-transformation bias, we implemented a combination of two methods: regression of observed on estimated values (Song, 2015) and Duan’s smearing estimate (Duan, 1983), described as the ROE-Duan method by Belitz and Stackelberg (2021). The final estimate of constituent concentration ($W_{ROE-Duan}$) on a given day in mg/l was derived from XGBest prediction ($Y_{\mathrm{XGBest}}$) using the following equations (Belitz and Stackelberg, 2021):

(1) $$Y_{ROE}=m_{ROE}\times Y_{XGBest}+b_{ROE}$$

(2) $$m_{ROE}=\frac{Cov\left(Y_{XGBest,TR},\;Y_{obs,TR}\right)}{Var\left(Y_{XGBest,TR}\right)}$$

(3) $$b_{ROE}=E\left(Y_{obs,TR}\right)-m_{ROE}\times E\left(Y_{XGBest,TR}\right)$$

(4) $$W_{ROE-\mathrm{Duan}}=exp\left(Y_{ROE}\right)\times E\left(exp\left(\varepsilon_{Y,TR}\right)\right)$$

where, $\varepsilon_{Y,TR}$ are the residuals of XGBest after correcting the predictions using the ROE method. $Y_{obs,TR}$ and $Y_{XGBest,TR}$ are the observed and predicted values of the training data in natural logarithmic scale.

#### 3.1.2. Model interpretations

Model inferences were derived using Shapely Additive Explanations (SHAP) (Lundberg and Lee, 2017) from XGBest to comprehensively evaluate each region’s constituent concentration and feature relationships. SHAP is a surrogate model interpretation approach that is based on the Shapley values (Shapley, 1953) from cooperative game theory and provides transparency in otherwise black-box ML models by quantifying the contribution of each feature to individual predictions. SHAP values estimate the marginal contribution of each feature to the model’s response. They describe the contribution of each feature (positive or negative) by distributing the difference between the predicted value and the mean observed value of the target variable among the features. The feature importance ranking for the entire model is determined by taking the mean of the absolute SHAP values for each feature of interest in the model, with higher values indicating greater relative importance. We used feature importance rankings and SHAP dependence plots to obtain regional (HUC-02 model scale) inferences for each water quality constituent (TN, TP, and TSS).

Further, we also used the SHAP values for ln(Q) to evaluate the local (site-specific) C-Q relationships at individual sites. Based on the methodology adopted by Meybeck and Moatar (2012), we split the relationship between ln(Q) and its SHAP values (corresponding to ln(C)) at the median discharge and estimated the slopes on each side. The slopes were considered statistically significant, indicating a chemodynamic pattern, if the *p*-value for the Pearson product-moment correlation was ≤0.05 (Moatar et al., 2017). The direction of the C-Q pattern (flushing or dilution) was determined by the sign of the slope of the trendline. C-Q relationships with a p-value >0.05 were classified as chemostatic.

### 3.2. Benchmarking

The XGBest predictive performance was benchmarked against the legacy United States Geological Survey (USGS) Load Estimator Tool (LOADEST) (Runkel et al., 2004), and the Weighted Regressions on Time, Discharge, and Season (WRTDS) tool (Hirsch et al., 2010).

#### 3.2.1. LOADEST

LOAD ESTimator, or LOADEST (Runkel et al., 2004), is a widely used method for estimating nutrient and sediment loads or concentrations in rivers and streams. It describes the relationship between discharge and solute or particulate concentration and develops a regression model to estimate constituent loads or concentrations. In addition to discharge and solute or particulate concentrations, it utilizes the time and day of sample collection information to account for seasonal and temporal variations in water quality. It provides an automated best model selection between nine regression equations, provided in Table S3 (SI), based on Akaike Information Criteria (AIC).

This study applied the LOADEST model using the automated best model selection method and Adjusted Maximum Likelihood Estimation (AMLE) individually at each site and for each available water quality parameter (TN, TP, and TSS) for predicting daily constituent concentration. Of the nine LOADEST equations (Table S3 in SI), Equation - S9, which includes coefficients for discharge, decimal time, as well as seasonality (sine and cosine functions of decimal time), was most frequently selected for predicting the three water quality parameters individually at each site (Fig. S2 in SI). Eqs. (S1), (S2), (S3), and (S5), which do not include the sine and cosine functions of decimal time (i.e., seasonality), were the least selected equations indicating the relevance of seasonality in predicting constituent concentrations.

#### 3.2.2. WRTDS

Weighted Regressions on Time, Discharge, and Season, or WRTDS, is a statistical method developed by Hirsch et al. (2010), that utilizes weighted regression over time, discharge, and season to generate daily estimates of constituent concentrations and loads. The weights are assigned to each data point in the observed water quality data based on its similarity to the estimation day in terms of time, discharge, and season. This weighting helps account for the variability and uncertainty in water quality observations. The regression equation implemented in the WRTDS model (Hirsch et al., 2010) is described as follows:

(6) $$ln\left(C_{i}\right)=\beta_{0,i}+\beta_{1,i}t_{i}+\beta_{2,i}ln\left(Q_{i}\right)+\beta_{3,i}sin\left(2\pi t_{i}\right)+\beta_{4,i}cos\left(2\pi t_{i}\right)+\varepsilon_{i}$$

where, $C_{i}$ is the constituent concentration on the day i (mg/l), t is the time of day i in decimal year, $Q_{i}$ is the observed discharge on the day i (cfs), $\beta_{0-4,i}$ are the model coefficients that vary smoothly over the model domain, and $\varepsilon_{i}$ is the unexplained variation on the day i.

The smoothing parameters in the WRTDS model used to estimate the overall weight for each data point include the half window length of ln (Q), trend, and season; these were set to 2.0, 10, and 0.5 respectively, as suggested by Hirsch et al. (2010), and were used in fitting the WRTDS model at each site to predict daily concentrations of TN, TP, and TSS using the *EGRET* R package (Hirsch et al., 2015).

### 3.3. Evaluation metrics

For the three modeling approaches (XGBest, LOADEST, and WRTDS), 20 % of the data from each water quality monitoring site were randomly withheld as an independent validation set, while the remaining 80 % were used for model training. To minimize potential bias from a single train-validation split, this random sub-setting was repeated 20 times, and models were re-run. The same training and validation sets were used across all three models to ensure a fair and consistent comparison of their performance.

Model performance was evaluated using Nash-Sutcliffe efficiency (NSE) (Nash and Sutcliffe, 1970), Kling-Gupta Efficiency (KGE) (Gupta et al., 2009), and % Flux bias (FBIAS) (Hirsch, 2014), across each water quality sampling site. We set the thresholds for satisfactory model performance for predicting daily scale constituent concentrations at individual to NSE > 0.3, KGE > 0.3, and FBIAS <±15 % for both nutrients and sediments. The NSE, KGE, and FBIAS values at each site are calculated as follows:

(7) $$NSE=1-\frac{\sum_{i=1}^{N}{\left(O_{i}\;-\;S_{i}\right)}^{2}}{\sum_{i=1}^{N}{\left(O_{i}\;-\;{\bar{O}}_{i}\right)}^{2}};-\infty <NSE\leq 1$$

(8) $$KGE=1-\sqrt{{\left(r-1\right)}^{2}+{\left(s-1\right)}^{2}+{\left(c-1\right)}^{2}};-\infty <KGE\leq 1$$

(9) $$FBIAS=\frac{\sum_{i=1}^{N}\left(O_{i}\;-\;S_{i}\right)\;\times \;Q_{i}}{\sum_{i=1}^{N}O_{i}\;\times \;Q_{i}}\times 100;-\infty <FBIAS\;<+\infty$$

where, $O_{i}$ and $S_{i}$ are the observed and simulated value of constituent concentration (mg/l) at time-step i,N is the total number of records, $Q_{i}$ is the observed discharge at time-step i,r is the correlation coefficient between observed and simulated values, s is the ratio of the standard deviation of simulated values to the standard deviation of observed values, and c is the ratio of the mean of simulated values to the mean of observed values.

## 4. Results and discussion

The observed mean daily concentrations of nutrients and sediments across the selected water quality monitoring sites, exhibit a large spatial and temporal variability over the 25-year study period (1996–2020). The sparsely sampled concentrations range from 0.1 to 3060 mg/L for TSS measured at 325 sites, 0.002 to 30.6 mg/L for TP at 432 sites, and 0.02 to 230 mg/L for TN at 380 sites. Section 4.1 discusses the results on the validity of study assumptions, followed by Section 4.2 that discusses the performance of the XGBest (bias-corrected) on predicting daily mean concentrations of TN, TP, and TSS, and compare its performance with the LOADEST and WRTDS models. Then, the regional (model scale) and local (site-specific) interpretations of XGBest using SHAP values are discussed in Section 4.3.

### 4.1. Assumptions evaluation

**Fig. 3.**
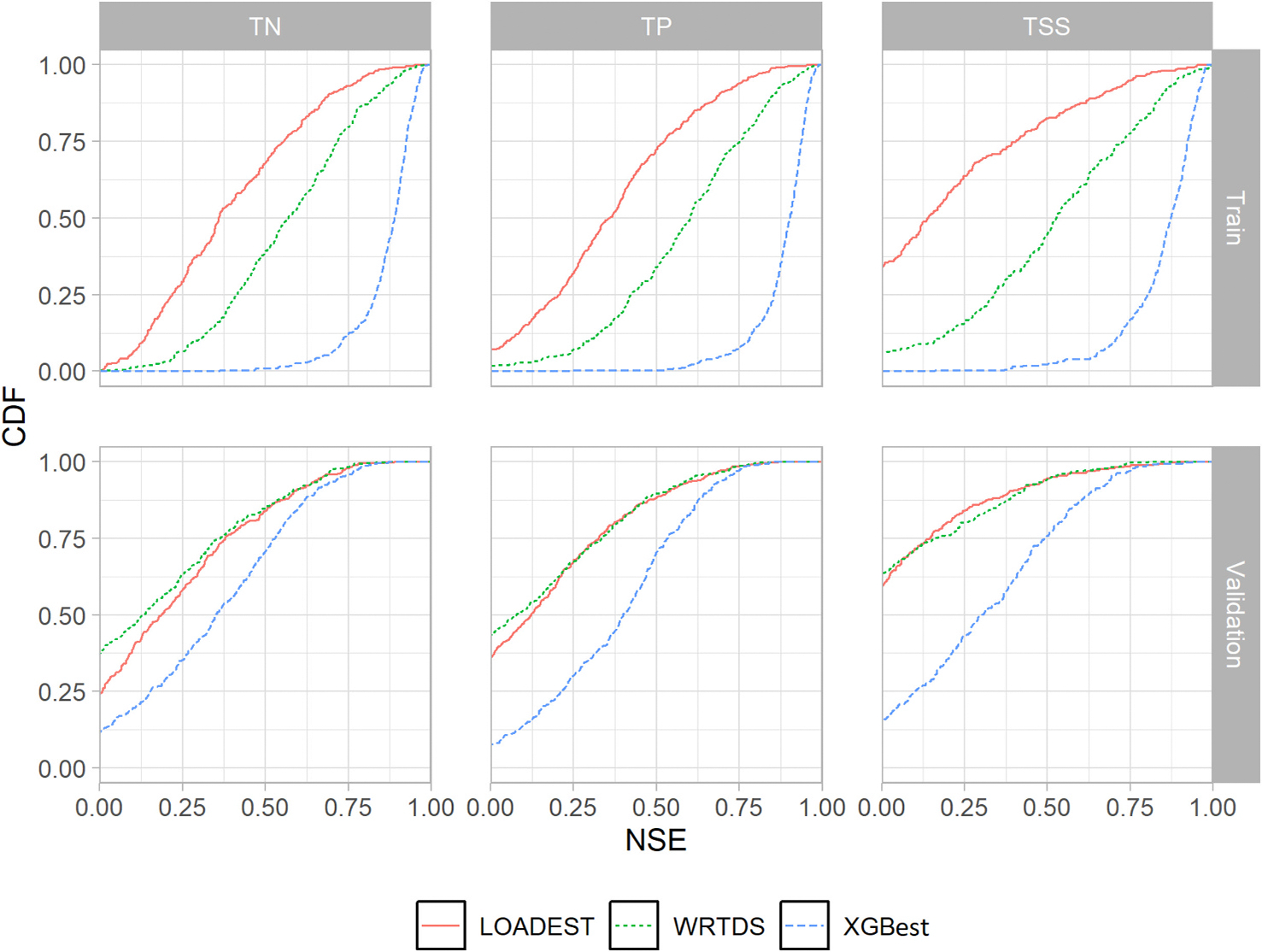
Comparison of the cumulative distribution of train and validation NSE values for the LOADEST, WRTDS, and XGBest across each water quality sampling site.

The XGBest approach was based on three key assumptions, discussed in the Introduction section. First, the approach involved developing a single prediction model for each constituent within a HUC02 hydrologic region by combining observed constituent concentration and discharge data for all the monitoring sites within the region. Prediction results, as shown in Fig. 3 and Table 2 and discussed in detail in Section 4.2, suggest that combining observed data helps overcome the challenge of limited data availability at individual sites to train prediction models. The XGBest predictions for constituent concentration at individual sites were found to be in better agreement with the observed data than those from LOADEST and WRTDS, supporting the validity of assumption I. Moreover, the ML based XGBest predictions showed significantly better agreement between predicted and observed datasets, validating assumption II, especially for the sites where LOADEST and WRTDS failed to converge or resulted in very low (< 0.3) or negative NSE values (Fig. 3). Section 4.3 provides a detailed analysis of the importance of static watershed attributes in predicting constituent concentrations, validating assumption III, which emphasizes the necessity of including static watershed attributes in water quality prediction models.

### 4.2. Model performance

The cumulative distribution of NSE values for predicting daily instream concentrations of TN, TP, and TSS using LOADEST, WRTDS, and XGBest across study sites in Fig. 3 shows a large heterogeneity in model performance across sites and water quality parameters. Overall, XGBest outperformed the LOADEST and WRTDS models for predicting daily concentrations across all three water quality parameters for most sites. XGBest showed the greatest improvement over the LOADEST and WRTDS models for predicting daily TSS concentrations. Both LOADEST and WRTDS models performed poorly for predicting TSS, with only 41 % and 37 % of the sites reporting validation set NSE greater than zero for LOADEST and WRTDS, respectively. In comparison, >85 % of the total sites reported NSE for TSS greater than zero in XGBest. Out of the 325 sites for TSS, the validation set NSE values for XGBest were higher for 78 % of the sites compared to 15 % for LOADEST and only about 7 % for WRTDS. However, for the sites where LOADEST and WRTDS had higher NSE than XGBest, the difference in their NSE values compared to XGBest was <0.2 for 74 % of the sites across all three constituents. In contrast, for sites where XGBest performed better, the difference in NSE values was higher than 0.6 NSE for 55 % of the stations.

For nutrients, XGBest achieved higher NSE values for approximately 77 % of the sites for both TN and TP. In contrast, LOADEST outperformed XGBest at 12 % of the sites for TN and 14 % of the sites for TP, while WRTDS performed best at only 11 % of the sites for TN and 9 % of the sites for TP. Out of 380 sites for TN and 432 sites for TP, about 88 % of the TN sites and 92 % of the TP sites showed positive NSE values with XGBest. For LOADEST, 75 % of the sites for TN and 64 % of the sites for TP had NSE values greater than zero. For WRTDS, only 63 % of the TN sites and 56 % of the TP sites reported positive NSE values.

Table 2 indicates the number of sites that fall within the satisfactory limits for each evaluation metric for the validation set (NSE > 0.3, KGE > 0.3, |FBIAS| < 15). XGBest had the highest number of sites within satisfactory limits compared to LOADEST and WRTDS based on NSE and KGE values for all three water quality parameters. However, both LOADEST and WRTDS reported more sites with FBIAS ≤ ±15 than XGBest. Although retransformation bias correction using the ROE-DUAN method yielded nearly unbiased estimates for the training set across most XGBest models, a comparable improvement was not seen for the validation set (Table S4 in SI). Also, the observed differences in FBIAS may have resulted from LOADEST and WRTDS applying bias correction to individual sites, whereas XGBest applies it collectively across all sites in the region. However, at sites where LOADEST and WRTDS outperformed XGBest, the difference in FBIAS was <10 % for 58 % and 50 % of the sites, respectively. Also, <1 % of all sites for all three water quality parameters reported FBIAS >50 % in XGBest, while 7 % of all sites for LOADEST and 13 % sites for WRTDS reported FBIAS >50 %. In addition, the large improvements in KGE and NSE values suggest the validity of XGBest for estimating water quality constituent concentrations and fluxes over the LOADEST and WRTDS models.

**Fig. 4.**
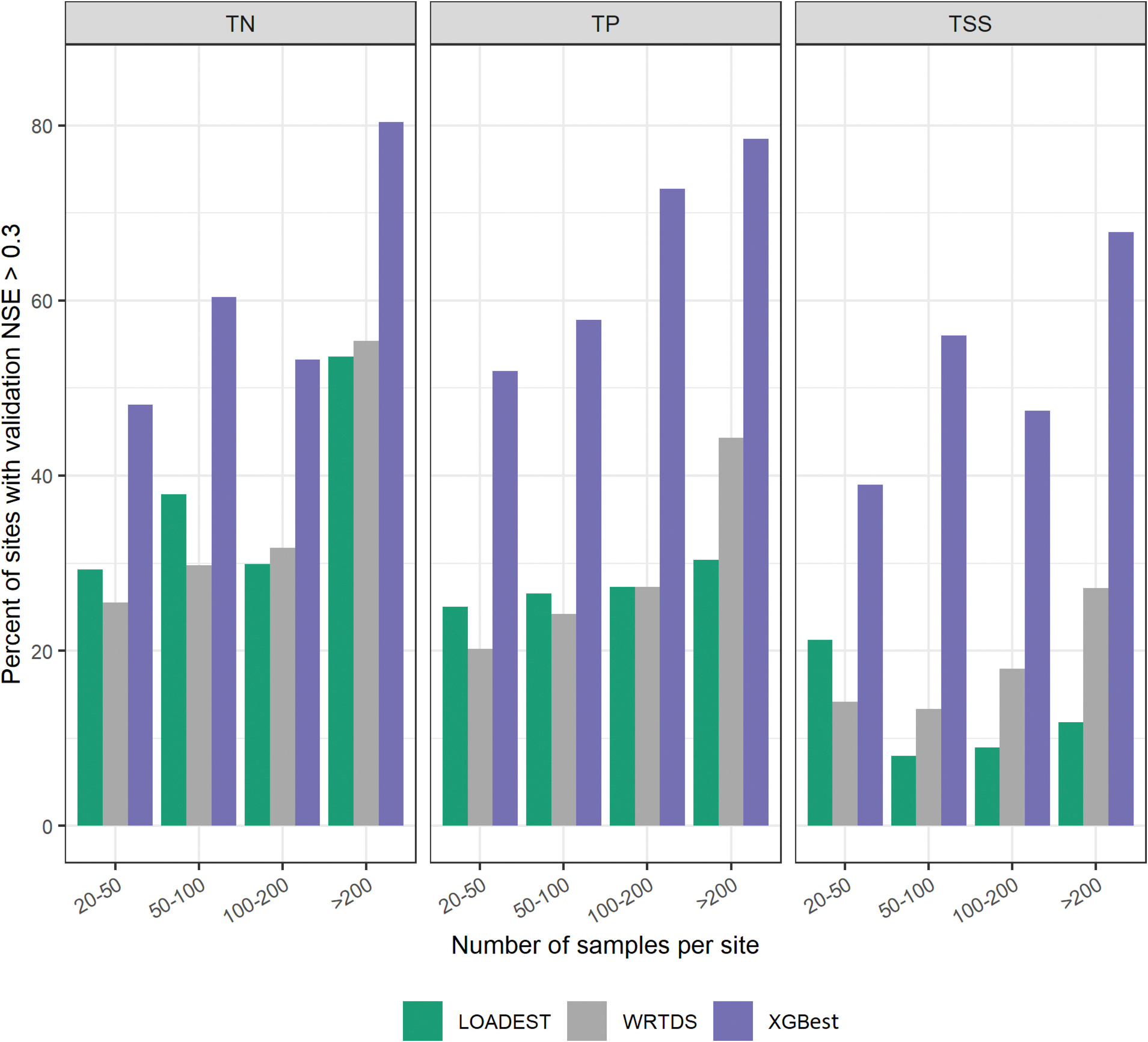
Comparison of the percentage of sites with validation NSE > 0.3 for LOADEST, WRTDS, and XGBest based on the number of sampling days.

Overall, 6 % of the sites for TN, 5 % for TP, and 9 % for TSS reported NSE < 0 in all three models. The number of available samples was <50 for 55 % of these sites and <100 for 87 % of these sites. Although the primary reason for this poor performance could be attributed to low sampling frequency (Fig. S1 in SI) and insufficient representation of constituent loads across various flow regimes and temporal scales, several other factors, such as large drainage areas, irrigation activities, point sources, and other anthropogenic activities may have impacted the prediction accuracy of the models. The performance of the WRTDS model in comparison to LOADEST was contingent upon the number of available water quality samples. The WRTDS approach is primarily developed for high frequency and long-term sites with >200 samples and 20 years of available data (Hirsch et al., 2010; Zhang and Hirsch, 2019). From this analysis, it is further evident that LOADEST may be preferred over WRTDS for sites with <50 samples, given the choice is only between these two approaches (Fig. 4). On the training set, WRTDS demonstrated higher performance relative to LOADEST (Fig. 3), which may suggest that WRTDS may be overfitting in sites with small number of training samples (Goswami et al., 2023). Fig. 4 illustrates the percentage of sites with an NSE value >0.3 in the validation set, categorized by the number of observed samples. Notably, WRTDS outperforms LOADEST at sites with >200 sampling days across all three parameters. However, XGBest consistently surpasses both WRTDS and LOADEST, irrespective of sampling days. This highlights the XGBest’s enhanced ability to accurately predict water quality parameters by leveraging observed data from multiple sites.

### 4.3. ML model interpretations

**Fig. 5.**
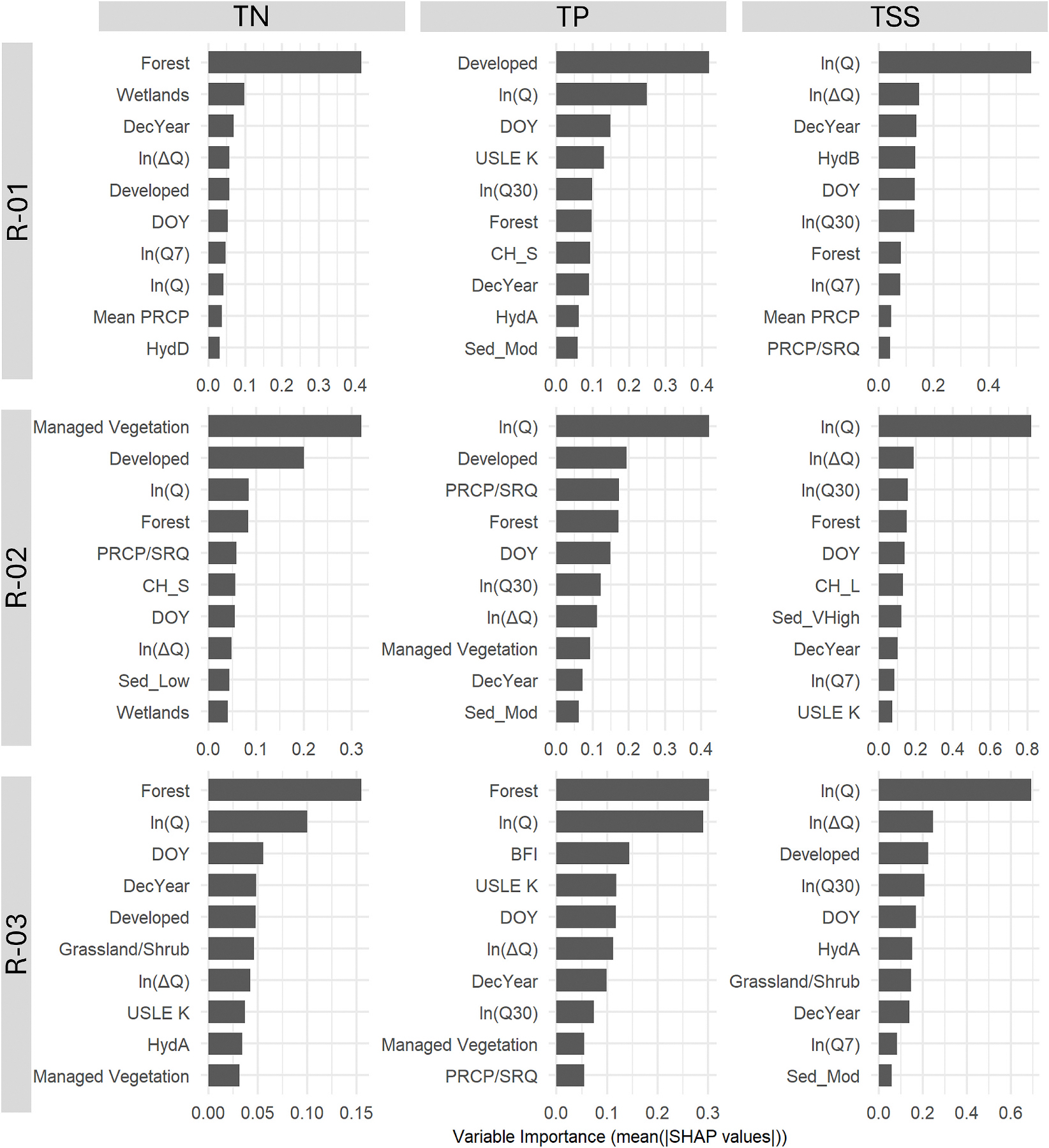
Relative importance of the ten most significant features in XGBest by hydrologic region and water quality parameter (TN, TP, and TSS) based on SHAP values.

The variable importance plots shown in Fig. 5 indicate high variability in the relative importance of watershed attributes, discharge, season, and time variables between water quality constituents and hydrological regions. For TSS, the discharge variables (ln(Q), ln(ΔQ), ln (Q7), and ln(Q30)) were all ranked within the top 10 features across all three regions, indicating a significant impact of both current and antecedent discharge conditions on TSS, with ln(Q) being the most important feature in all three regions for TSS. However, for both TN and TP, several watershed attributes were ranked relatively higher than discharge. This can be attributed to a larger spatial variance in instream nutrients between watersheds relative to temporal variance within each watershed. The variance in geomeans of water quality samples between sites for TN in R-01 and R-02 is more than sixteen times larger than the median variance in samples within sites. In comparison, the variance in geomeans between sites for TSS is only 0.96 times the median variance in samples within sites in R-01, 0.27 times in R-02, and 0.1 times in R-03, which can also be directly attributed to the higher relative importance ranking for ln(Q) for TSS.

Among the watershed attributes, land cover characteristics (specifically % of Forest and Developed area) were frequently ranked as the most important predictors of TN and TP. More than 50 % of the study watersheds in R-01 and R-02 are predominantly forested, with a forest cover fraction >0.5 (Fig. S3 in the SI). Many previous studies have also indicated land cover to be the most important predictor of water quality constituents as they can directly affect nutrient retention and removal (Behrouz et al., 2022; Maloney et al., 2022; Sheikholeslami and Hall, 2023). Maloney et al. (2022) also found forest and developed land cover to be the most important features in predicting in-stream biological conditions in the Chesapeake Bay watershed. We found forest cover negatively correlated to its SHAP value for both TN and TP in all three regions (Figs. S4–S6 in SI), indicating that increasing forest cover in watersheds may limit nutrient delivery to the streams due to organic matter accumulation (Bellmore et al., 2018). This inverse relationship may also stem from reduced erosion in forested areas compared to disturbed land covers (Corbett et al., 1997). Contrastively, SHAP values of % developed area were generally negative in undeveloped watersheds and positive for urban watersheds for all water quality parameters and HUC02 regions, indicating a larger contribution in nutrient and sediment concentrations from urban landscapes.

**Fig. 6.**
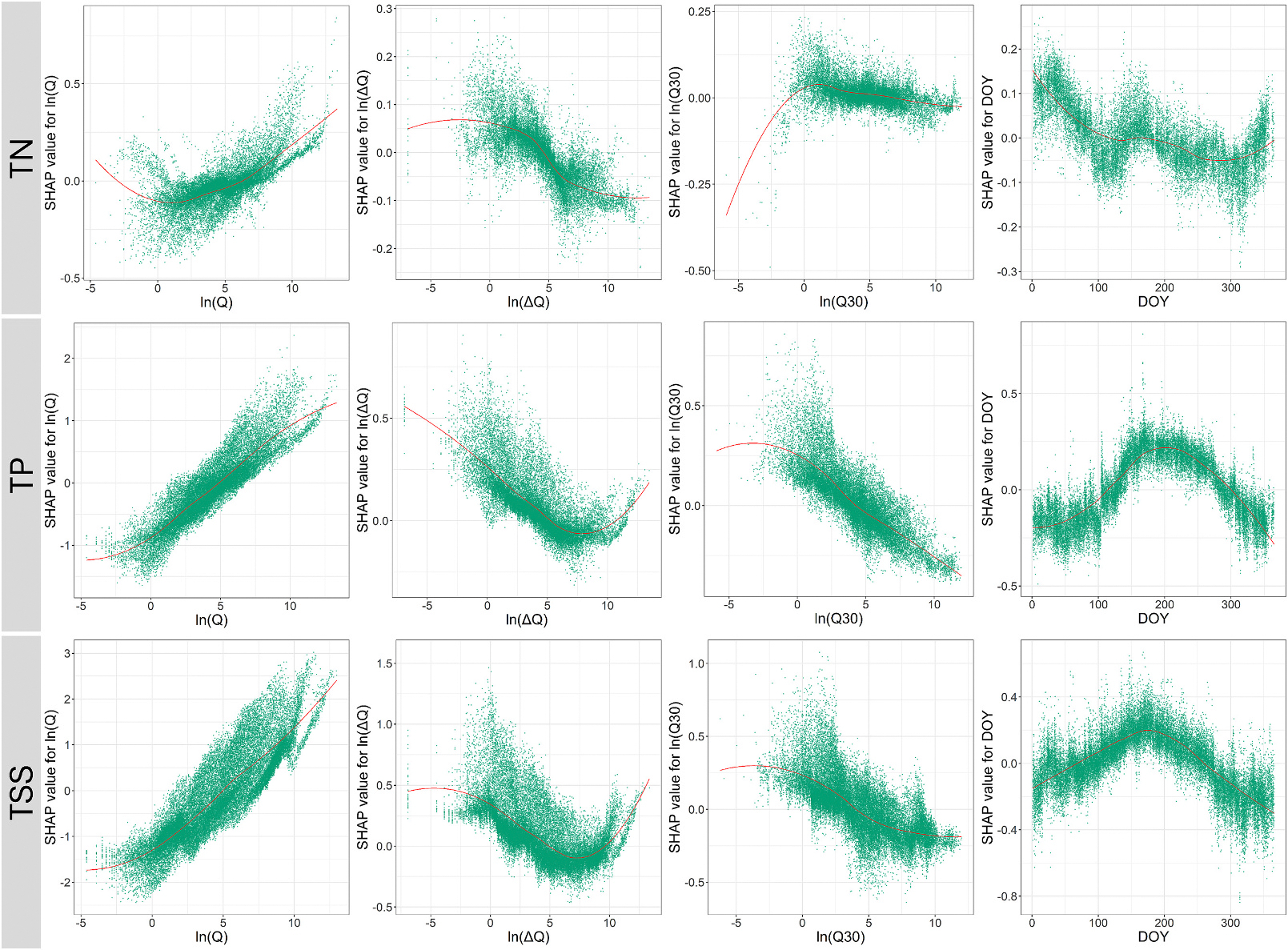
SHAP dependence plots for the logarithm of daily discharge (ln(Q)), 1-day lagged discharge (ln(ΔQ)), 30-day rolling mean discharge (ln(Q30)), and day of the year (DOY) values for TN, TP, and TSS in R-02 (Mid-Atlantic Region). The smooth line shows the trend in SHAP values, derived using the Locally Estimated Scatterplot Smoothing (LOESS) method (Cleveland, 1979).

The SHAP dependence plots of ln(Q) for R-02 in Fig. 6 illustrate a generally increasing contribution of ln(Q) to TP and TSS concentrations with increasing ln(Q), which is representative of the dominance of flushing effects for TP and TSS in most watersheds. For TN, SHAP dependence plots exhibit larger heteroscedasticity in SHAP values of ln (Q), indicating multiple C-Q modalities for TN across different watersheds. SHAP dependence plots for the 1-day lagged discharge (ln(ΔQ)) and its relative importance in predicting constituent concentrations reflect the hysteresis effects in the C-Q relationships. The high relative importance of ln(ΔQ) in predicting TSS may also suggest the large difference in the performance of XGBest in comparison to LOADEST and WRTDS, which do not account for antecedent flow conditions. For TP and TSS, an increase in 30-day rolling mean discharge (ln(Q30)) is generally associated with decreased nutrient and sediment concentrations (Fig. 6), which is indicative of the effects of instream dilution (Isles, 2024).

Further, Day of the Year (DOY), representative of seasonal trends in water quality constituent concentrations, was ranked within the top 5 predictors in eight of the nine XGBest models. Both TP and TSS show similar sinusoidal trends in SHAP values, with positive SHAP values between June and late October (late spring to early fall). These periods also coincide with the region’s highest average monthly temperatures and precipitation levels, indicating elevated total phosphorus (TP) and total suspended solids (TSS) concentrations due to increased surface runoff events. However, for TN, SHAP values were highest during the beginning of the year, which can be attributed to reduced plant uptake during winter months. Groundwater contribution may also serve as a source of TN during winter months when runoff is reduced (Lee et al., 2012). Additionally, instream denitrification rates typically decline in winter due to lower stream temperatures and snow cover, which could lead to an increase in TN during this period (Duff et al., 2002). A slight increase in DOY’s SHAP values in early fall indicates the increased agricultural activities, including manure applications and grazing during this period.

**Fig. 7.**
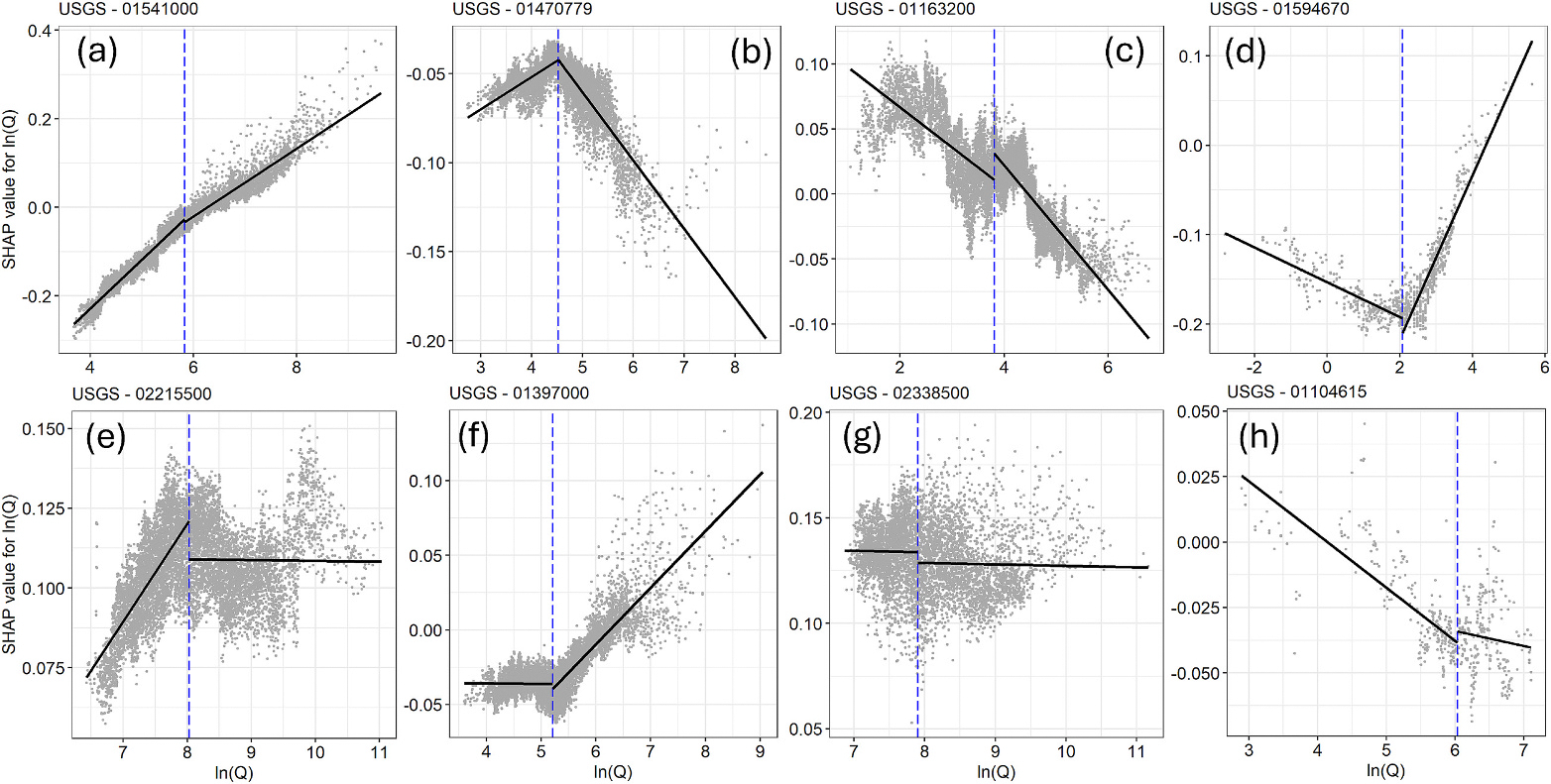
Observed eight C-Q patterns for TN in order of frequency of occurrence obtained from SHAP dependence plots of ln(Q) at individual sites: (a) flushing; (b) flushing until median discharge, then dilution; (c) dilution; (d) dilution until median discharge, then flushing; (e) flushing until median discharge, then chemostatic; (f) chemostatic until median discharge, then flushing; (g) chemostatic; (h) dilution then chemostatic. The vertical blue line indicates the natural logarithm of median discharge.

SHAP values can also be applied for model inferences at the local (site-specific) scale. Therefore, it is possible to discern the C-Q relationships by visualizing the dependence plot of SHAP values from estimated constituent concentrations over each site. The SHAP values for ln(Q) isolate the main and interaction effects of ln(Q) on ln(C) from the influences from temporal, seasonal, or antecedent flow variations, giving a more accurate representation of the C-Q relationship. For both TP and TSS, the concentration and discharge showed a flushing effect above and below median discharge for almost all sites in the study area (99 % for TSS and 96 % for TP), indicating a large increase in TP and TSS loads during high-flow events. Two additional C-Q patterns for TSS and TP were identified and are presented in Fig. S7 in SI. However, TN exhibited eight distinct C-Q patterns when split by the median discharge, illustrated in Fig. 7. The flushing pattern was the most dominant (78 % of the sites). “Flushing until median discharge, then dilution pattern” and the “dilution only pattern” each accounted for 6.5 % of the sites. The “dilution until median discharge, then flushing pattern” was found in 5 % of the sites. The remaining 4 % of the sites exhibited a chemostatic pattern on at least one side of the median discharge. These patterns are indicators of the biological and hydrological functioning of the watershed (Moatar et al., 2017; Saha et al., 2023). Identifying the relationships between the distinct concentration-discharge (C-Q) patterns and watershed attributes to identify the underlying reasons for these behaviors is a potential avenue for investigation but is beyond the scope of this study.

Future work could also explore the validity of the XGBest for making predictions in ungaged basins (PUB). This study did not explore such scenarios due to the significant influence of anthropogenic activities at many selected sites, which can markedly alter constituent concentrations in hydrologically similar watersheds. These alterations complicate assessments in the absence of comprehensive data on point sources and agricultural practices. Additionally, future work will focus on incorporating uncertainty estimation within the XGBest framework and comparing its performance against established approaches, such as the WRTDS Bootstrap Test (Hirsch et al., 2015). The recent applications of HAWQS in regions such as India, South Africa, Brazil, and Ukraine highlights the broader applicability of this approach for global water quality modeling efforts. Overall, the large-scale implementation of this study and its assimilation into HAWQS can support decision-making for sustainable watershed management practices, mitigating risks associated with eutrophication, harmful algal blooms, and sediment-driven habitat degradation that affect both ecological resilience and public health.

## 5. Conclusion

This study successfully developed and implemented the ML-based XGBest tool to predict daily instream concentrations and fluxes of TN, TP, and TSS over 499 water quality monitoring sites within three HUC02 hydrological regions in the eastern United States. The finding demonstrated that combining the sparsely sampled data from all sites within a hydrologic region to train the prediction model and incorporating physical watershed attributes along with discharge and seasonality matrices in prediction models significantly enhances water quality predictions. The XGBest outperformed LOADEST and WRTDS in predicting daily instream sediment and nutrient concentrations at individual sampling sites across all three study regions. Moreover, feature importance analysis using SHAP revealed that including the watershed attributes as explanatory variables in the XGBest models was critical in determining the spatial variability in constituent concentrations, with land cover and antecedent discharge emerging as key predictors. Furthermore, SHAP dependence plots at individual sites revealed that while TP and TSS exhibited dominant flushing patterns in the C-Q relationship, TN displayed a more complex range of patterns. Overall, this study presents XGBest as a robust, scalable, and reliable tool for water quality predictions. By effectively integrating diverse watershed characteristics and hydrological metrics, XGBest offers substantial improvements over traditional methods, making it a valuable resource for environmental monitoring and management efforts. Additionally, this study provides predictions on daily in-stream constituent concentrations and fluxes, along with performance evaluation statics, for all eighteen hydrologic regions in the lower conterminous United States using the methodology presented in the study, distributed through open-source data repositories and HAWQS.

## Supplementary Material

Supplement1

## Figures and Tables

**Table 1:** Description of static and dynamic features used in the XGBest model.

Category	Feature	Description

Discharge	ln(Q)	Natural log of daily average discharge (cfs)
	ln(ΔQ)	Natural log of 1-day lagged discharge (cfs)
	ln(7dQ)	Natural log of 7-day rolling average discharge (cfs)
	ln(30dQ)	Natural log of 30-day rolling average discharge (cfs)
Time	DOY	Day of the year of observed discharge
	DecYear	Year of observed discharge, expressed as a decimal
Land cover	Developed	Fraction of watershed area, developed
	Forest	Fraction of watershed area, forested
	Wetland	Fraction of watershed area, wetland
	Grassland/Shrub	Fraction of watershed area, Grassland and Shrubland
	Managed-Vegetation	Fraction of watershed area, cultivated crops, and Hay/Pasture
Hydrology	Mean PRCP	Mean annual precipitation (mm)
	SRQ	Mean annual surface runoff estimated from HAWQS (mm)
	BFI	Baseflow Index
Soil	HydA	Fraction of watershed area, soil hydrologic class A (high infiltration rates)
	HydB	Fraction of watershed area, soil hydrologic class B (moderate infiltration rates)
	HydC	Fraction of watershed area, soil hydrologic class C (slow infiltration rates)
	HydD	Fraction of watershed area, soil hydrologic class D (very low infiltration rates)
Loading potential^a^	Slope	Average watershed slope (%)
	USLE K	Erodibility factor (Wischmeier and Smith, 1978)
	Sed_VHigh	Fraction of watershed area, Very High sediment class
	Sed_High	Fraction of watershed area, High sediment class
	Sed_Mod	Fraction of watershed area, Moderate sediment class
	Sed Low	Fraction of watershed area, Low sediment class
	Sed_Vlow	Fraction of watershed area, Very Low sediment class
Channel	CH_L	Channel Length (m)
	CH_W	Channel Width (m)
	CH_S	Channel Slope (m/m)
	CH_D	Channel Depth (m)

a For detailed information on sediment class, please refer to Table S1 in SI.

*cfs* = cubic feet per second.

**Table 2:** Number of sites meeting the satisfactory performance criteria for LOADEST, WRTDS, and XGBest.

Par	Model	Number of sites meeting acceptable criteria		
		NSE criteria (NSE ≥ 0.3)	KGE criteria (KGE ≥ 0.3)	Flux Bias criteria (FBIAS ≤ ±15)

TP	LOADEST	117	249	365
	WRTDS	120	227	331
	XGBest	278	319	278
TN	LOADEST	135	203	371
	WRTDS	125	211	359
	XGBest	220	269	355
TSS	LOADEST	44	105	138
	WRTDS	56	112	156
	XGBest	163	203	116

## Data Availability

The codes required to reproduce the results are available at - https://github.com/GEM-TAMU/XGBest-WQ-Prediction. This study presents results on three HUC02 regions only, however, the required data and outputs for all 18 hydrologic regions (1479 TN sites; 2025 TP sites; 1614 TSS sites) in the lower conterminous U.S. are available at – doi:10.18738/T8/8X7QMA.

## HIGHLIGHTS

- XGBest enhances understanding of spatial-temporal variability in water quality.
- XGBest outperformed LOADEST and WRTDS for in-stream water quality prediction.
- Land cover was the most effective predictor of in-stream nutrient concentrations.
- SHAP values revealed a flushing concentration-discharge pattern in most watersheds.
- SHAP analysis reveals critical environmental factors in water quality dynamics.
