# Supplementary material for: Enhancing prediction and inference of daily in-stream nutrient and sediment concentrations using an extreme gradient boosting based water quality estimation tool - XGBest
Source: Sci Total Environ. Author manuscript; Available in PMC 2026 Feb 1. (PMC11833449; doi:10.1016/j.scitotenv.2025.178517)
Supplement: Supplement1 [file NIHMS2053122-supplement-Supplement1.docx]

Supplementary Information (SI)

Enhancing Prediction and Inference of Daily In-stream Nutrient and Sediment Concentrations using an Extreme Gradient Boosting based Water Quality Estimation Tool - XGBest

Shubham Jain^1,2^, Arun Bawa^2, *^, Katie Mendoza^2^, Raghavan Srinivasan^2^, Rajbir Parmar^3^, Deron Smith^3^, Kurt Wolfe^3^, and John M. Johnston^3^

^1^Water Management and Hydrological Science, Texas A&M University, College Station, TX, USA

^2^Texas A&M AgriLife Research, Blackland Research & Extension Center, Temple, TX, USA

^3^Office of Research and Development, United States Environmental Protection Agency, Athens, GA, USA

^*^ Corresponding author – Arun Bawa (Email – arun.bawa@ag.tamu.edu)


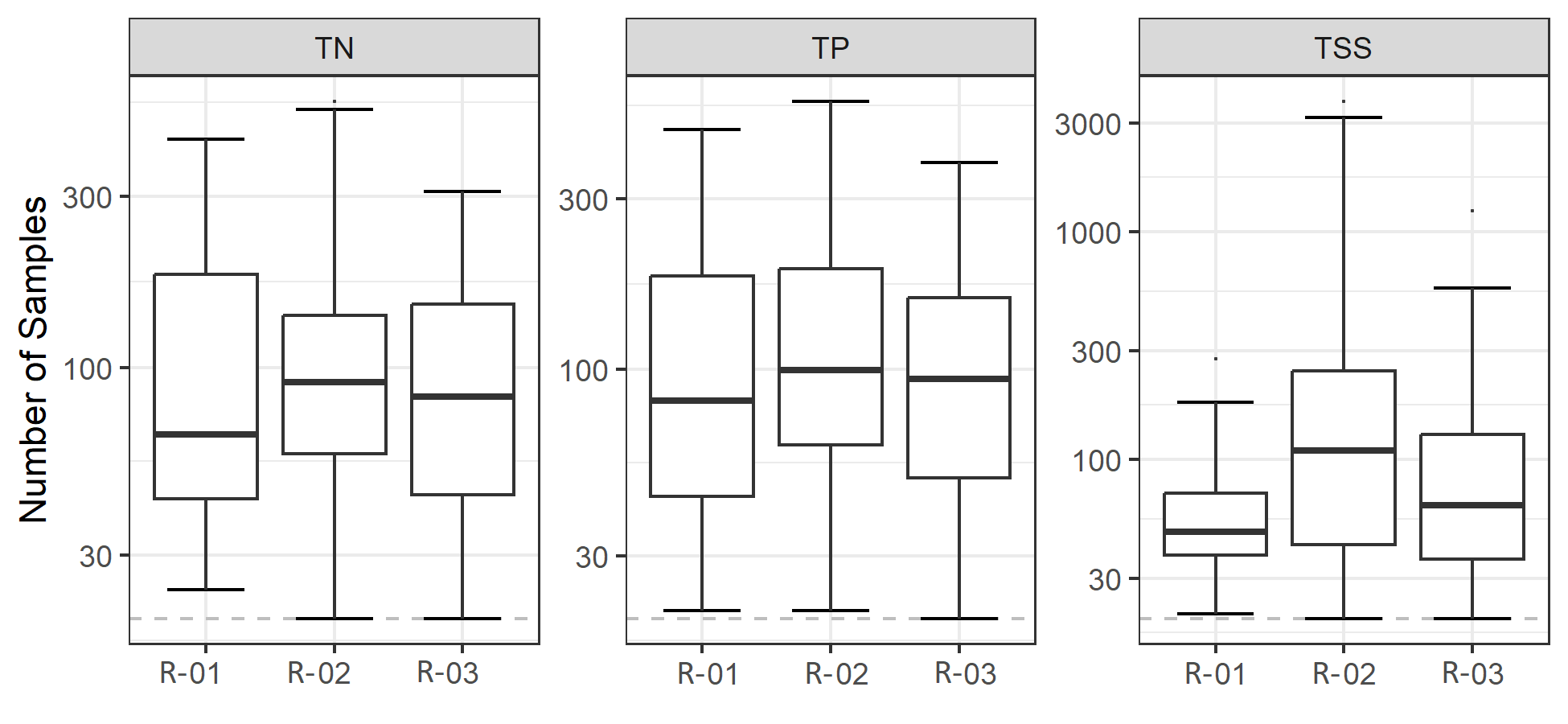


Figure S1. Boxplots of number of samples collected at each study site between 1996 and 2020 for each water quality parameter (TN, TP, TSS) categorized by the hydrologic region. The horizontal dashed line at 20 samples shows the minimum sample criteria for site selection.


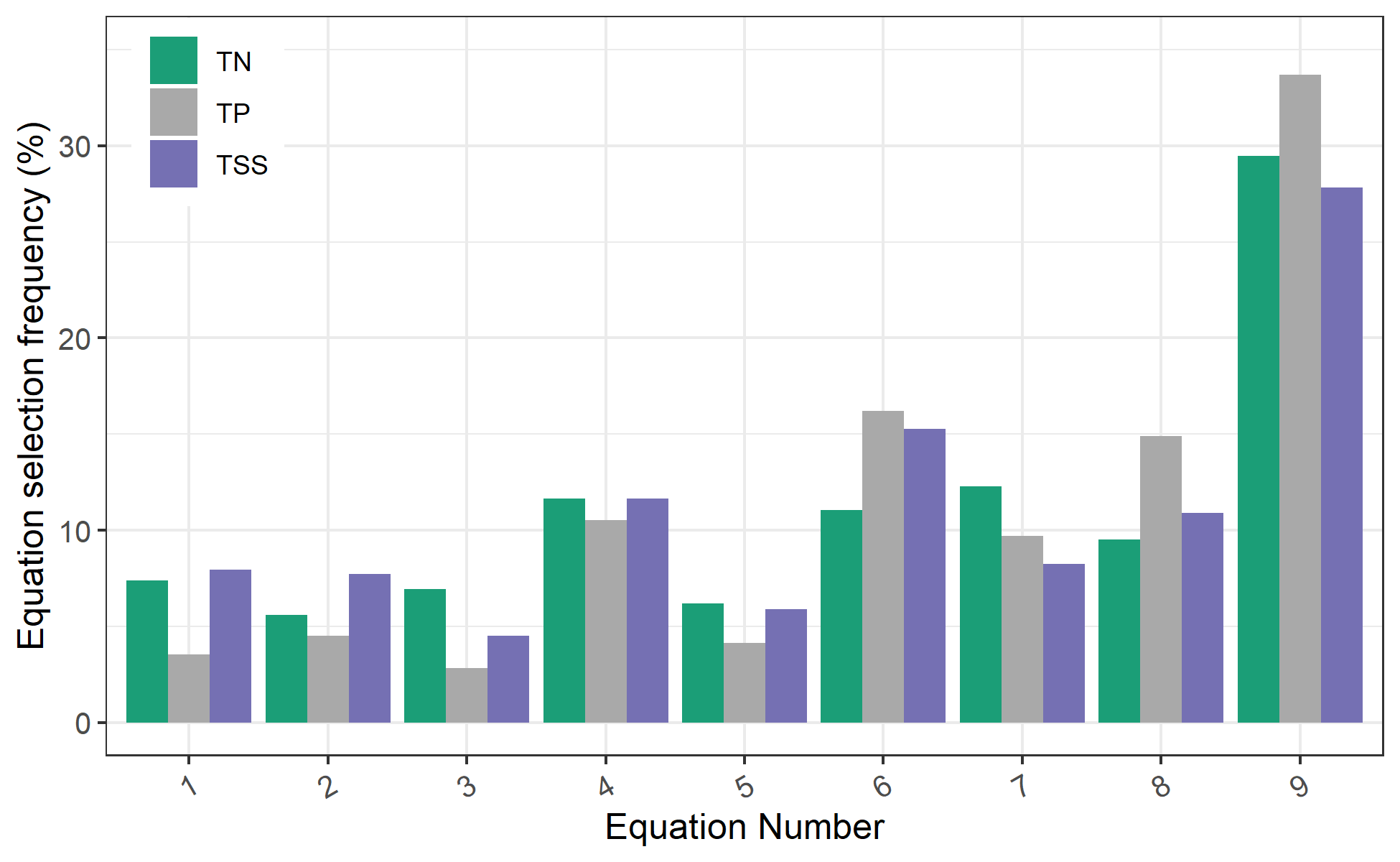


Figure S2. The percentage of instances for each of the nine LOADEST equations was selected using the automated model selection approach across all study sites and 20 train-validation iterations.


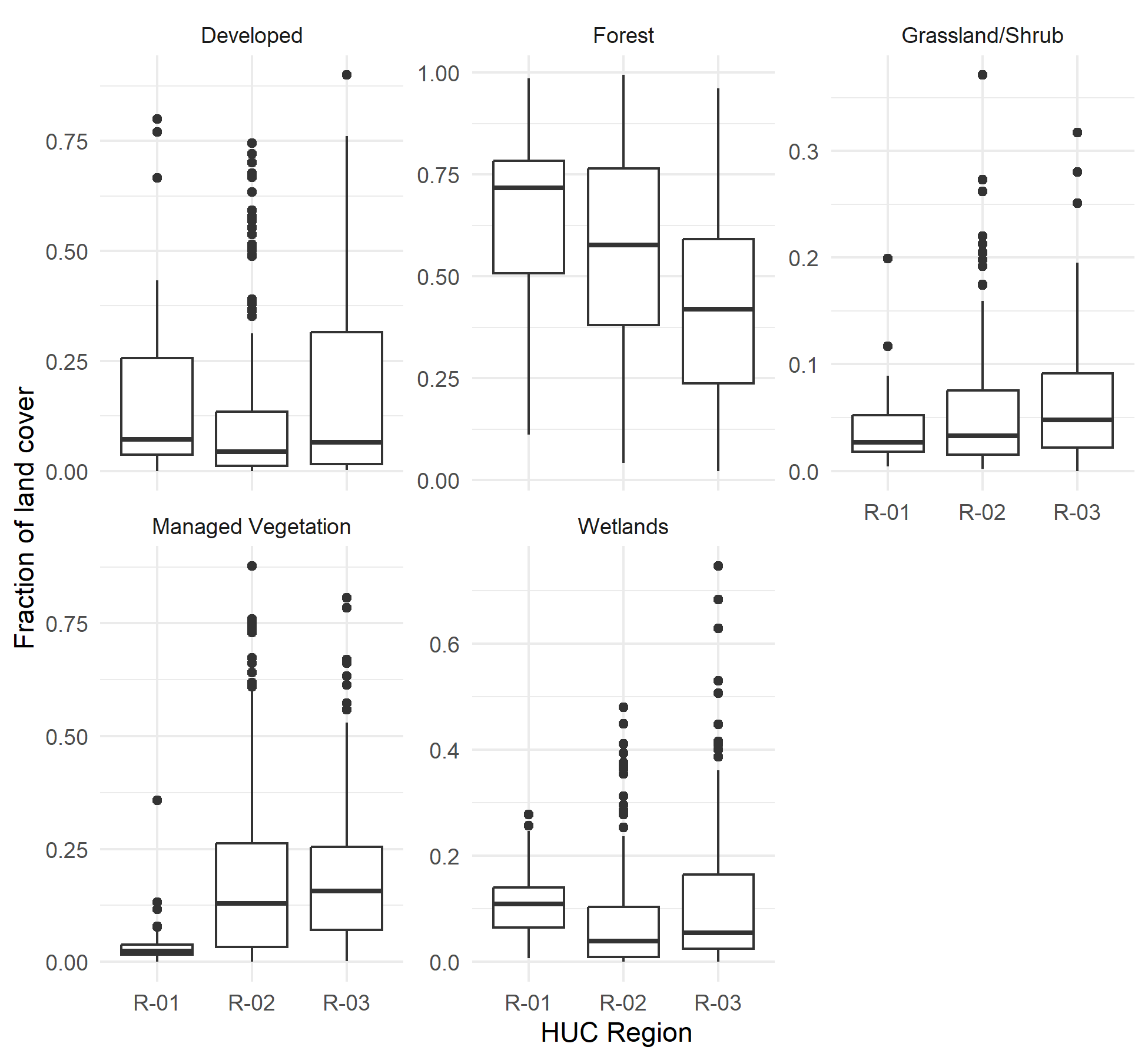


Figure S3. Boxplots of the land cover type fractions for study watersheds across each hydrologic region.


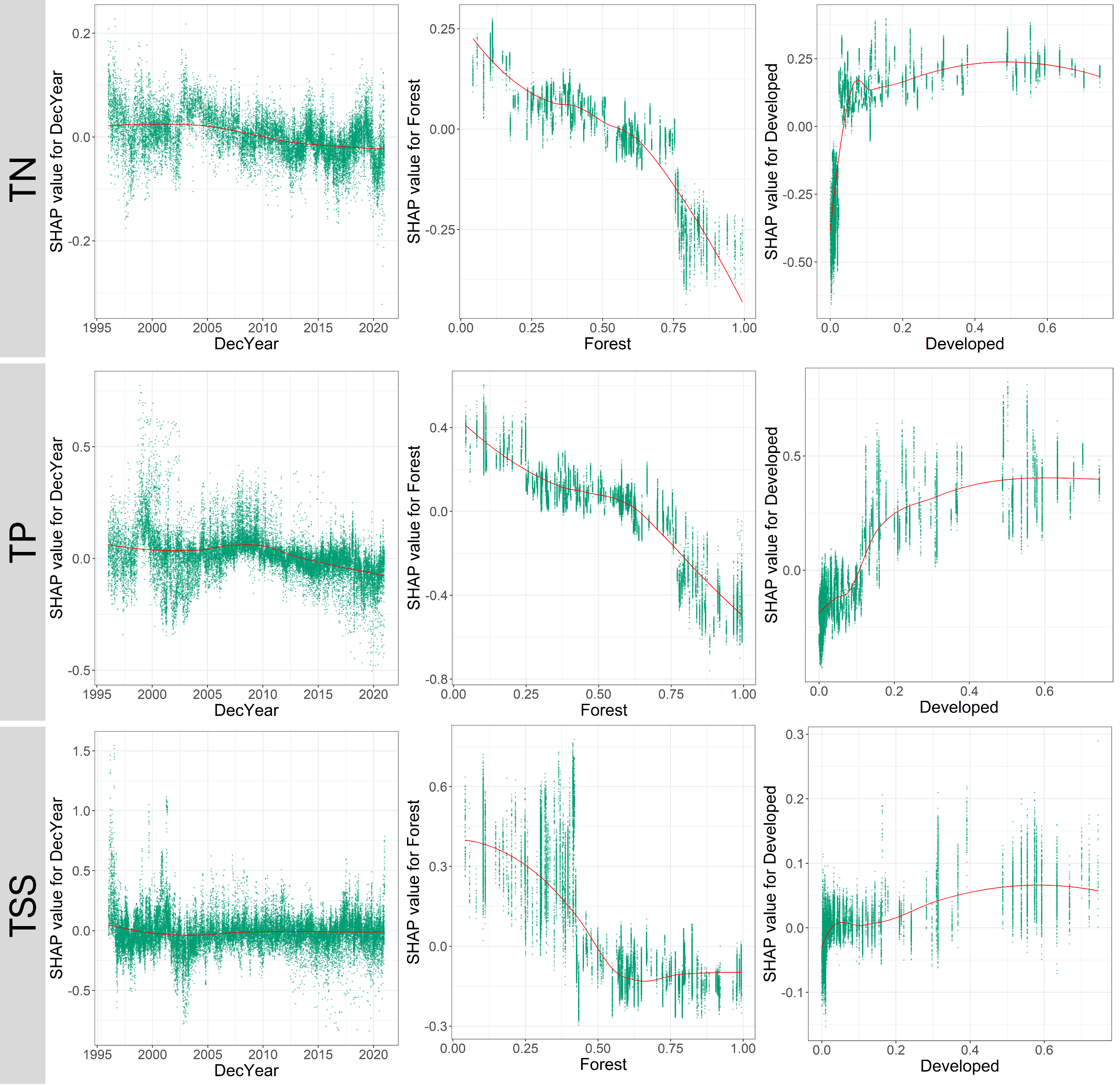


Figure S4. SHAP dependence plots for the DecYear, % Forest area, and % Developed area for TN, TP, and TSS in R-02 (Mid-Atlantic Region).


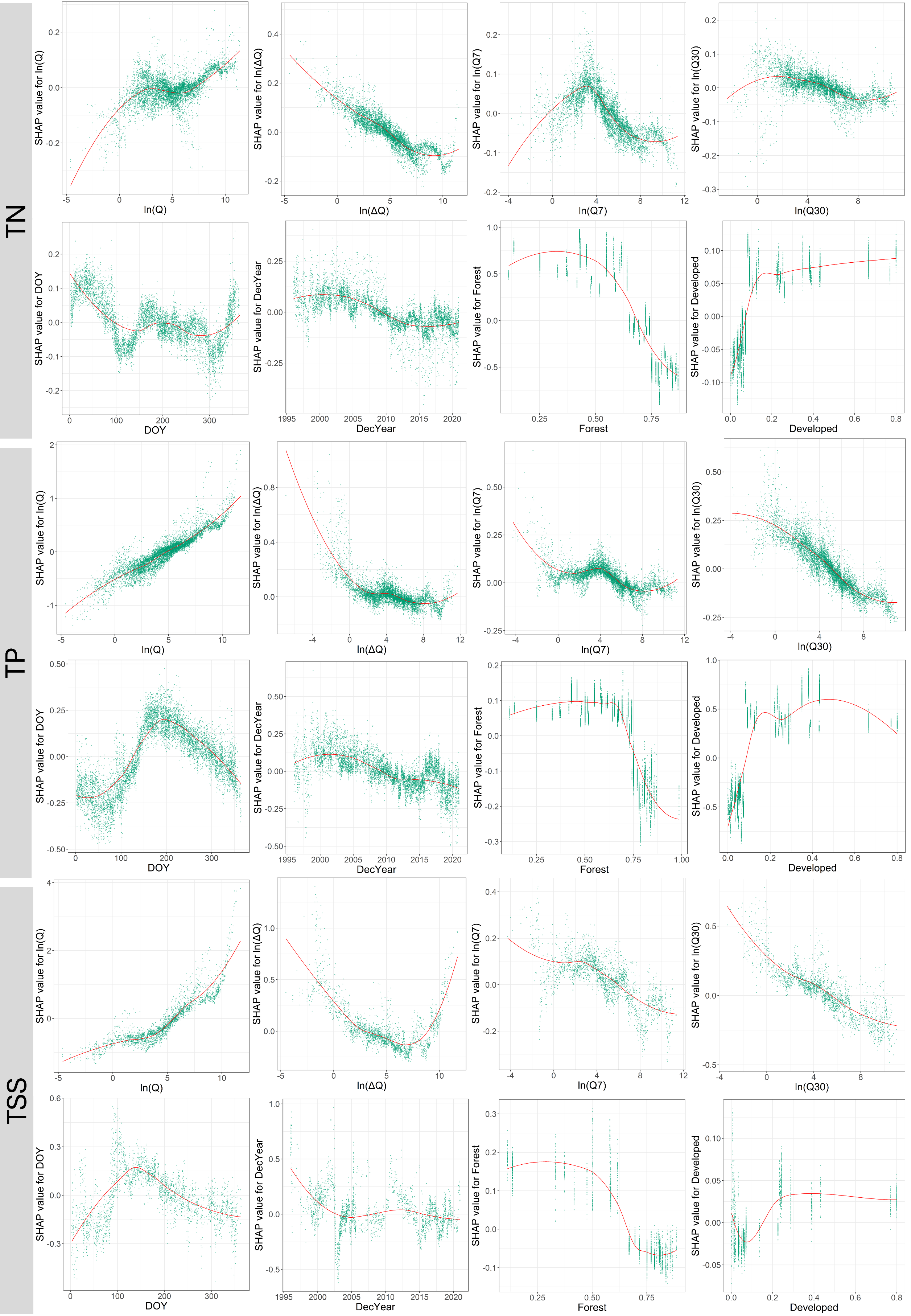


Figure S5. SHAP dependence plots for the logarithm of daily discharge (ln(Q)), 1-day lagged discharge (ln(ΔQ)), 30-day rolling mean discharge (ln(Q30)), day of the year (DOY), Decimal Year (DecYear), % Forest area, and % Developed area for TN, TP, and TSS in R-01 (New England Region).


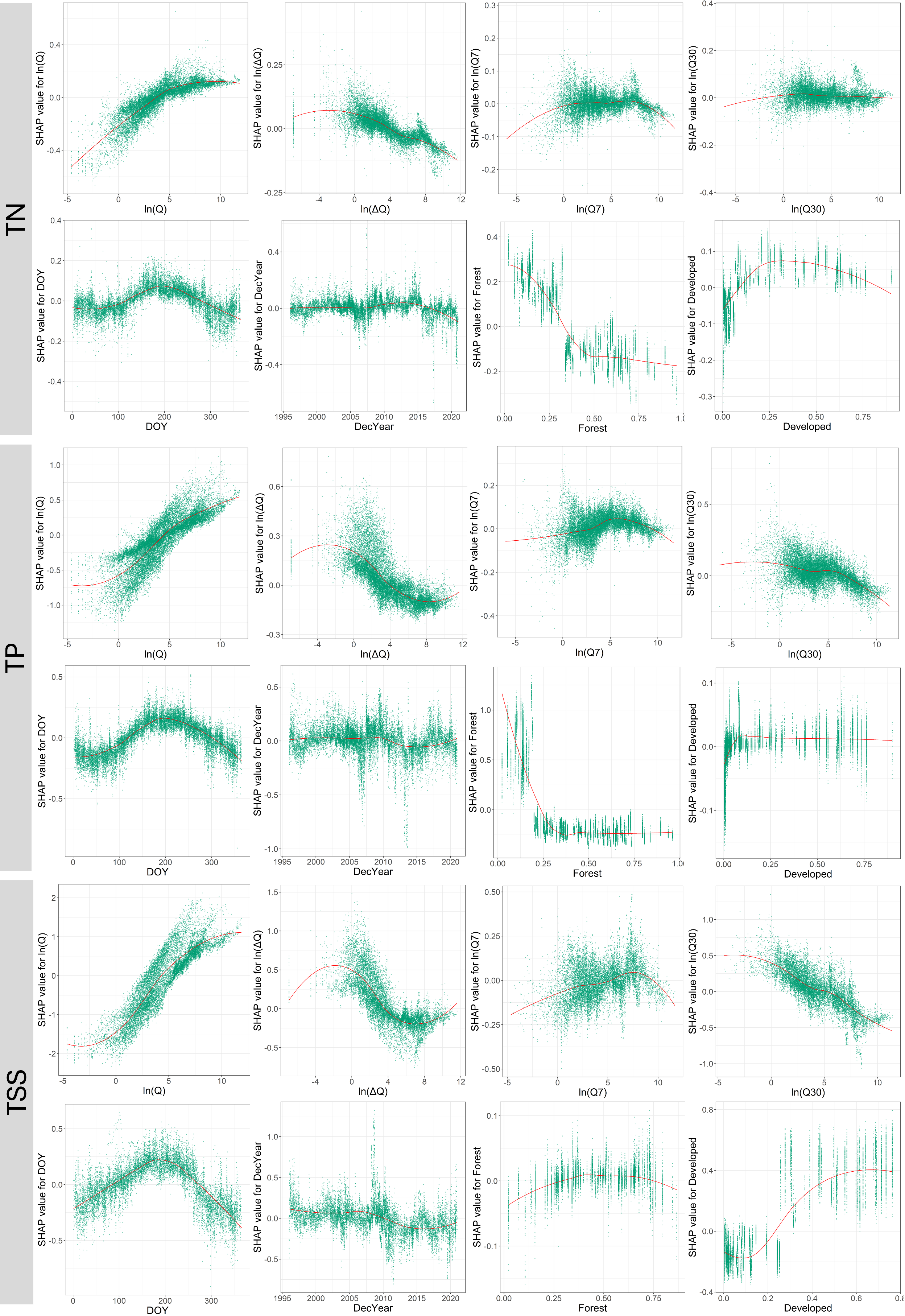


Figure S6. SHAP dependence plots for the logarithm of daily discharge (ln(Q)), 1-day lagged discharge (ln(ΔQ)), 30-day rolling mean discharge (ln(Q30)), day of the year (DOY), Decimal Year (DecYear), % Forest area, and % Developed area for TN, TP, and TSS in R-03 (South Atlantic-Gulf Region).


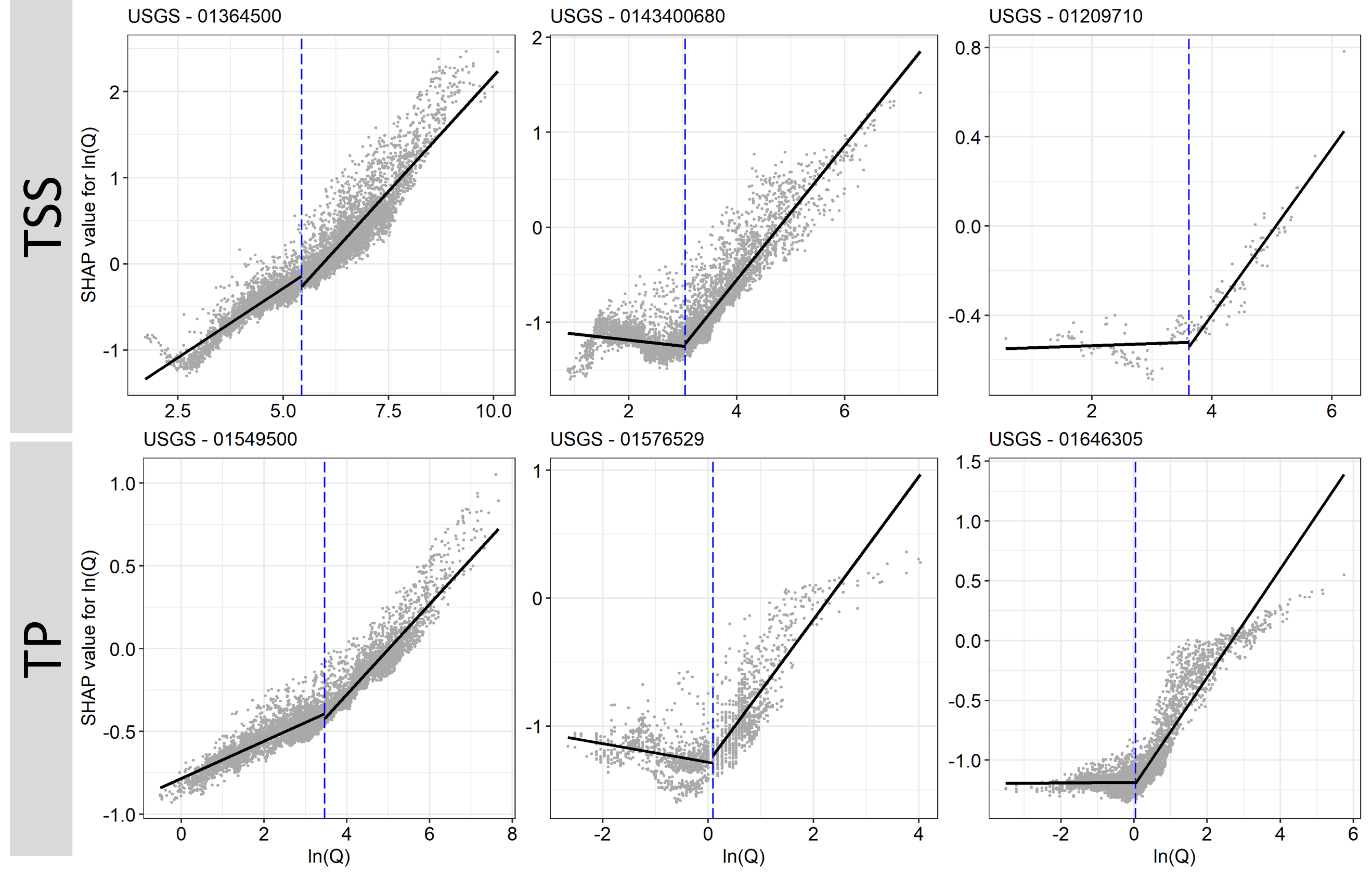


Figure S7. Examples of identified C-Q patterns for TP and TSS using SHAP dependence plots. The vertical blue line indicates the natural logarithm of median discharge.

Table S1. Description of sediment loading classes used as watershed attributes in XGBest.

| **Curve number** | **Slope** | **Sediment loading class** |
| --- | --- | --- |
| > 85 | > 8% | Very High |
|  | 2 – 8% |  |
| > 85 | 0 – 2% | High |
| 70 - 85 | > 8% |  |
|  | 2 – 8% |  |
| 70 - 85 | 0 – 2% | Moderate |
| 50 - 70 | > 8% |  |
|  | 2 – 8% |  |
| 50 – 70 | 0 – 2% | Low |
| 35 – 50 | > 8% |  |
|  | 2 – 8% |  |
| 35 – 50 | 0 – 2% | Very Low |
| < 35 | > 8% |  |
|  | 2 – 8% |  |
|  | 0 – 2% |  |
| Curve number: > 85 very high runoff potential; 70-85 high runoff potential; 50-70 high runoff potential; 35-50 high runoff potential; <35 high runoff potential.  Slope: >8% Steep terrain; 2-8% Moderately sloping terrain; 0-2% flat to gently sloping terrain. | | |

Table S2. Potential ranges of the hyperparameters used for grid search and the selected values of the final nine XGBest models without train-validation data splitting.

| Hyperparameters | Potential values | TN | | | TP | | | TSS | | |
| --- | --- | --- | --- | --- | --- | --- | --- | --- | --- | --- |
|  |  | HUC02 - Region | | | | | | | | |
|  |  | R-01 | R-02 | R-03 | R-01 | R-02 | R-03 | R-01 | R-02 | R-03 |
| *max_depth* | 5:20 | 9 | 16 | 11 | 14 | 12 | 14 | 9 | 17 | 11 |
| *eta* | 0.01-0.3 | 0.09 | 0.04 | 0.04 | 0.02 | 0.02 | 0.03 | 0.05 | 0.02 | 0.04 |
| *gamma* | 0-0.2 | 0.05 | 0.16 | 0.05 | 0.09 | 0.03 | 0.13 | 0.01 | 0.04 | 0.08 |
| *subsample* | 0.6-0.9 | 0.68 | 0.61 | 0.68 | 0.71 | 0.88 | 0.74 | 0.64 | 0.83 | 0.64 |
| *colsample_bytree* | 0.5-0.8 | 0.79 | 0.71 | 0.69 | 0.78 | 0.67 | 0.78 | 0.79 | 0.69 | 0.73 |
| *min_child_weight* | 1:10 | 4.00 | 5.00 | 5.00 | 7.00 | 9.00 | 8.00 | 8.00 | 10.00 | 6.00 |
| *max_delta_step* | 1:10 | 2.00 | 4.00 | 4.00 | 8.00 | 4.00 | 7.00 | 8.00 | 7.00 | 10.00 |

Table S3. Equations used in the LOADEST automated selection method. The best equation is selected using Adjusted Maximum Likelihood Estimation (AMLE) and used to predict constituent concentrations.

| Eq. No. | Equation |
| --- | --- |
| S1 | $lnC=a_{0}+a_{1}lnQ$ |
| S2 | $lnC=a_{0}+a_{1}lnQ+a_{2}lnQ^{2}$ |
| S3 | $lnC=a_{0}+a_{1}lnQ+a_{2}dtime$ |
| S4 | $lnC=a_{0}+a_{1}lnQ+a_{2}\sin\left( 2\pi dtime \right)+a_{3}cos(2\pi dtime)$ |
| S5 | $lnC=a_{0}+a_{1}lnQ+a_{2}lnQ^{2}+a_{3}dtime$ |
| S6 | $lnC=a_{0}+a_{1}lnQ+a_{2}lnQ^{2}+a_{3}\sin\left( 2\pi dtime \right)+a_{4}\cos\left( 2\pi dtime \right)$ |
| S7 | $lnC=a_{0}+a_{1}lnQ+a_{2}\sin\left( 2\pi dtime \right)+a_{3}\cos\left( 2\pi dtime \right)+a_{4}dtime$ |
| S8 | $lnC=a_{0}+a_{1}lnQ+ a_{2}lnQ^{2}+a_{3}\sin\left( 2\pi dtime \right)+a_{4}\cos\left( 2\pi dtime \right)+a_{5}dtime$ |
| S9 | $lnC=a_{0}+a_{1}lnQ+ a_{2}lnQ^{2}+a_{3}\sin\left( 2\pi dtime \right)+a_{4}\cos\left( 2\pi dtime \right)+a_{5}dtime+a_{6}dtime^{2}$ |
| Where, $\ln\left( C \right)$ is the log of constituent concentration, $a_{0-6}$are model coefficients, $\ln\left( Q \right)$ is log of streamflow and $dtime$ is decimal time. | |

Table S4. Train and validation % Flux Bias (FBIAS) for pre- and post-bias correction in the nine XGBest models using the ROE-DUAN method for a single train-validation split iteration.

| Par |  | Train FBIAS | | | Validation FBIAS | | |
| --- | --- | --- | --- | --- | --- | --- | --- |
|  | HUC02 | Pre | Post | % Reduction | Pre | Post | % Reduction |
| TN | R-01 | -0.8 | 0 | 100.0 | -4.5 | -0.8 | 82.2 |
|  | R-02 | -0.5 | 0 | 100.0 | -2.5 | -0.5 | 80.0 |
|  | R-03 | -5.6 | -1.8 | 67.9 | -7.4 | -5.6 | 24.3 |
| TP | R-01 | -4.5 | -0.5 | 88.9 | -9.4 | -4.5 | 52.1 |
|  | R-02 | -6.9 | -0.4 | 94.2 | -15.7 | -6.9 | 56.1 |
|  | R-03 | -7.2 | 1.3 | 81.9 | -15 | -7.2 | 52.0 |
| TSS | R-01 | -27.7 | 11.6 | 58.1 | -38.1 | -27.7 | 27.3 |
|  | R-02 | -11.8 | 1.2 | 89.8 | -34.2 | -11.8 | 65.5 |
|  | R-03 | -14.7 | -0.3 | 98.0 | -36.1 | -14.7 | 59.3 |
